# A Comprehensive Review of the Immunomodulatory Effects of Vitamin D in Sepsis

**DOI:** 10.7759/cureus.53678

**Published:** 2024-02-05

**Authors:** Abhinav Ahuja, Sachin Agrawal, Sourya Acharya, Sunil Kumar

**Affiliations:** 1 Medicine, Jawaharlal Nehru Medical College, Datta Meghe Institute of Higher Education & Research, Wardha, IND

**Keywords:** clinical outcomes, inflammation, immune response, immunomodulation, vitamin d, sepsis

## Abstract

Sepsis remains a critical global health challenge characterised by a dysregulated immune response to infection, leading to systemic inflammation and organ dysfunction. This review examines the immunomodulatory effects of Vitamin D in sepsis, focusing on its regulation of immune cell function, modulation of cytokine production, and enhancement of antimicrobial responses. While the potential of Vitamin D as an adjunctive therapy in sepsis management is evident, challenges such as variability in Vitamin D status, uncertainties regarding optimal dosages and patient heterogeneity, and potential adverse effects require careful consideration. The review highlights the implications for future research and clinical practice, emphasising the need for standardised measurement protocols, elucidation of optimal supplementation strategies, and integration of Vitamin D assessments into routine care. Despite the complexities, Vitamin D emerges as a promising avenue for personalised interventions in sepsis, necessitating ongoing research collaboration and evidence-based guidelines to harness its full therapeutic potential and improve clinical outcomes.

## Introduction and background

Sepsis, a life-threatening condition resulting from the body's dysregulated response to infection, continues to pose a significant global health challenge. Defined by organ dysfunction due to a dysregulated host response to infection, sepsis remains a leading cause of morbidity and mortality in hospitals worldwide. Despite advancements in medical science, unravelling the intricate immunomodulatory responses underlying sepsis remains a complex task, and identifying targeted therapeutic interventions is paramount [1]. Sepsis represents a critical clinical syndrome characterised by a systemic inflammatory response to infection. What begins as an infection localised to a specific organ or tissue can rapidly escalate, triggering a cascade of events that lead to widespread inflammation, organ dysfunction, and, in severe cases, septic shock [2]. The clinical manifestations of sepsis are diverse, ranging from fever and increased heart rate to organ failure, and its incidence continues to rise, making it a substantial burden on healthcare systems globally [2].

The immune response in sepsis is a double-edged sword. While an effective immune response is crucial for combating infections, an exaggerated or dysregulated immune reaction can contribute to tissue damage and organ dysfunction. Understanding the delicate balance between pro-inflammatory and anti-inflammatory responses during sepsis is essential for devising targeted therapeutic strategies. Immunomodulation, the modulation of the immune system to restore balance, emerges as a promising avenue for addressing the complex pathophysiology of sepsis [3]. Vitamin D, traditionally recognised for its role in calcium homeostasis and bone health, has garnered increasing attention for its non-skeletal effects, particularly in modulating the immune system. As research unfolds, the immunomodulatory properties of vitamin D have become a focal point in investigating its potential therapeutic role in various diseases, including sepsis. This review aims to explore and synthesise the current evidence surrounding Vitamin D's immunomodulatory effects in sepsis, shedding light on its mechanisms of action and potential clinical implications. By delving into the intersection of Vitamin D and sepsis, we seek to contribute to the growing body of knowledge to refine therapeutic approaches for this challenging clinical syndrome.

## Review

Immunomodulatory mechanisms of Vitamin D

Regulation of Immune Cell Function

T lymphocytes: Vitamin D's impact on T lymphocytes, crucial components of the adaptive immune system, is notable for its regulatory effects on T cell differentiation. Specifically, Vitamin D has been observed to exert a profound influence by inhibiting the excessive activation of pro-inflammatory T helper 1 (Th1) cells. Moreover, it promotes the development of regulatory T cells (Tregs), establishing a delicate balance crucial in sepsis. In this condition, dysregulated Th1 responses contribute to tissue damage. By modulating T cell differentiation, Vitamin D offers a promising avenue for preventing an overwhelming inflammatory response, potentially mitigating the severity of sepsis [4].

B lymphocytes: The role of Vitamin D in B lymphocytes, the key mediators of humoral immunity, is gaining recognition. Research suggests that Vitamin D may regulate B cell proliferation and antibody production. Vitamin D may contribute significantly to maintaining immune homeostasis during sepsis by creating an environment conducive to appropriate B cell responses. This dual impact on B lymphocytes holds promise for preventing both immunodeficiency and hyperactivation, potentially addressing a crucial aspect of the dysregulated immune response observed in septic conditions [5].

Macrophages: Macrophages, central players in the innate immune response and integral to sepsis pathogenesis, are subject to modulation by Vitamin D. Studies indicate that Vitamin D influences macrophage function by enhancing their phagocytic activity while concurrently suppressing the production of pro-inflammatory cytokines. This dual regulatory action is pivotal in balancing adequate pathogen clearance and preventing excessive inflammation. Vitamin D's modulation of macrophages offers a potential therapeutic avenue in sepsis, where an imbalanced immune response can lead to organ dysfunction. The fine-tuning of macrophage function by Vitamin D may mitigate the risk of sepsis-induced organ dysfunction, representing a nuanced approach to immune modulation in this critical clinical syndrome [6].

Modulation of Cytokine Production

Vitamin D's impact on the immune system extends to the precise regulation of cytokine production, representing a pivotal facet of the inflammatory response. Through a series of intricate mechanisms, Vitamin D has demonstrated its capacity to modulate the synthesis of critical cytokines, orchestrating a delicate balance between pro-inflammatory and anti-inflammatory signals. Notably, studies have consistently shown that Vitamin D possesses the ability to suppress the production of pro-inflammatory cytokines, including interleukin-6 (IL-6) and tumour necrosis factor-alpha (TNF-α), both crucial players in the inflammatory cascade [7]. In the context of sepsis, where an unbridled cytokine storm can lead to systemic inflammation and contribute to extensive tissue damage, the regulatory influence of Vitamin D assumes critical importance. Vitamin D's capacity to temper the overproduction of pro-inflammatory cytokines may act as a regulatory brake, preventing the escalation of inflammation to severe stages. Concurrently, Vitamin D promotes the production of anti-inflammatory cytokines, such as interleukin-10 (IL-10), which contributes to establishing an anti-inflammatory milieu. This intricate balancing act by Vitamin D becomes particularly significant in mitigating the severity of sepsis and holds the potential to minimise associated organ damage [8]. By fine-tuning the cytokine response, Vitamin D emerges as a promising modulator of the inflammatory cascade in sepsis. Its ability to shift the balance from excessive pro-inflammatory signals toward a more controlled and regulated immune response highlights its therapeutic potential in attenuating the severity of sepsis, thereby offering a novel avenue for intervention in the complex pathophysiology of this critical clinical syndrome [9].

Influence on Innate Immunity

Vitamin D is pivotal in shaping the body's primary defence mechanisms against pathogens, profoundly influencing innate immunity- the frontline protection against invading microorganisms. Vitamin D's ability to enhance the antimicrobial activity of various innate immune cells, including neutrophils and monocytes, is noteworthy. This enhancement is particularly crucial in the early stages of the immune response during sepsis, where a rapid and effective defence is paramount to prevent the unchecked progression of infection [10]. One of the fundamental mechanisms through which Vitamin D reinforces innate immunity is by facilitating the production of antimicrobial peptides, with cathelicidin being a prominent example. These peptides play a crucial role in the body's ability to fend off microbial invaders, acting as natural antibiotics that target a broad spectrum of pathogens. Vitamin D's role in promoting the synthesis of cathelicidin and other antimicrobial peptides strengthens the innate immune response, bolstering the host's defences against potential pathogens [11]. The significance of Vitamin D's multifaceted influence on innate immune cells becomes particularly evident in the context of sepsis management. In the early stages of sepsis, when the body requires an immediate and efficient response to curb the spread of infection, Vitamin D's ability to enhance antimicrobial activity and peptide production could be instrumental. This underscores Vitamin D's potential as a critical player in fortifying the body's defences against pathogens, presenting valuable insights into potential therapeutic interventions in sepsis. Understanding and harnessing these innate immune modulatory properties of Vitamin D may pave the way for innovative approaches to enhance the host's resilience against microbial challenges, with implications for managing sepsis and related infectious conditions [12]. Table 1 describes Vitamin D's immunomodulatory effects in sepsis.

**Table 1:** Vitamin D's immunomodulatory effects in sepsis

Immunomodulatory aspect	Effects of Vitamin D
Regulation of immune cell function	Modulation of T lymphocytes: Inhibits excessive activation of pro-inflammatory Th1 cells and promotes regulatory T cell development.
	Impact on B lymphocytes: regulates B cell proliferation and antibody production.
	Modulation of Macrophages: enhances phagocytic activity and suppresses pro-inflammatory cytokine production.
Modulation of cytokine production	Suppression of pro-inflammatory cytokines: IL-6, TNF-α.
	Promotion of anti-inflammatory cytokines: IL-10.
Influence on innate immunity	Enhancement of antimicrobial activity of innate immune cells: neutrophils, monocytes.
	Facilitation of antimicrobial peptide production, e.g., cathelicidin.

Sepsis: pathophysiology and immune response

Overview of Sepsis Pathophysiology

Sepsis is characterised by a dysregulated host response to infection, leading to systemic inflammation and organ dysfunction. The sepsis pathophysiology involves a complex interplay between the invading pathogen and the host's immune system. Initially, an infection triggers a localised immune response to eliminate the pathogen. However, in sepsis, this response becomes dysregulated, resulting in a cascade of events that can lead to widespread tissue damage and organ failure [13]. The progression of sepsis involves various stages, including infection, systemic inflammatory response syndrome (SIRS), sepsis, severe sepsis, and septic shock. The transition from localised infection to systemic inflammation and organ dysfunction is marked by a dysregulated immune response, with an imbalance of pro-inflammatory and anti-inflammatory signals contributing to the pathophysiological changes observed in sepsis [14].

Dysregulated Immune Response in Sepsis

An exaggerated pro-inflammatory phase characterises the immune response in sepsis, and a subsequent anti-inflammatory phase is collectively called a "cytokine storm." Pro-inflammatory cytokines, such as interleukin-1 (IL-1), interleukin-6 (IL-6), and tumour necrosis factor-alpha (TNF-α), are released in abundance, contributing to systemic inflammation. This initial surge in pro-inflammatory signals can lead to endothelial dysfunction, increased vascular permeability, and impaired microcirculation, contributing to organ dysfunction [15]. Conversely, the anti-inflammatory response, marked by increased anti-inflammatory cytokines like interleukin-10 (IL-10), may result in immune suppression and an impaired ability to clear the underlying infection. This delicate balance between pro-inflammatory and anti-inflammatory responses is disrupted in sepsis, leading to immune dysregulation that significantly impacts patient outcomes [16].

Implications for Therapeutic Interventions

Understanding the dysregulated immune response in sepsis is crucial for developing targeted therapeutic interventions. Given the multifaceted nature of sepsis pathophysiology, a one-size-fits-all approach is unlikely to be effective. Therapeutic strategies aim to modulate the immune response, restore immune balance, and prevent or mitigate organ dysfunction [14]. The conventional approaches include broad-spectrum antibiotics to target the underlying infection and supportive care to address organ dysfunction. However, the growing recognition of immune dysregulation in sepsis has sparked interest in immunomodulatory therapies. This is where Vitamin D's potential comes into focus, as its ability to modulate immune cell function, cytokine production, and innate immunity aligns with the intricate immunopathology observed in sepsis [17].

Vitamin D in sepsis: experimental studies

Key Findings on Immunomodulatory Effects of Vitamin D in Sepsis

Numerous studies have explored the correlation between Vitamin D and sepsis, shedding light on its potential immunomodulatory effects. Research indicates that a deficiency in Vitamin D may contribute to sepsis and is associated with increased susceptibility to infections [18]. This deficiency has also been linked to a higher risk of severe sepsis or septic shock in patients [19]. Vitamin D has demonstrated the potential to prevent both community- and hospital-acquired infections and reduce the severity of sepsis and subsequent organ dysfunction [20]. Animal experiments have shown that Vitamin D can alleviate sepsis-induced acute lung injury by downregulating ER stress [21]. Although the clinical impact of Vitamin D on sepsis is an ongoing area of research, evidence suggests that supplementing with Vitamin D during intensive care unit stays is linked to improved outcomes in critically ill patients with sepsis [21]. However, translating these findings to critically ill patients with sepsis may pose challenges, and further research is necessary to comprehend the clinical implications [20].

Limitations and Gaps in Current Research

Despite the promising discoveries, there are limitations and gaps in the existing research regarding the immunomodulatory effects of Vitamin D in sepsis. Studies investigating the relationship between Vitamin D insufficiency and sepsis in critical illness have employed varied designs and yielded mixed results, posing challenges in arriving at definitive conclusions [20]. The potential impact of Vitamin D on other physiological systems complicates efforts to isolate its specific connection to sepsis in critically ill patients, introducing complexity to the research [20]. While animal studies have shown positive outcomes, translating these findings to human studies and demonstrating mortality benefits in sepsis through Vitamin D presents challenges [20]. Despite indications of a potential link between Vitamin D and sepsis, further research is imperative to address the current limitations and gaps in knowledge. This research aims to ascertain the clinical implications of Vitamin D supplementation in sepsis.

Challenges and Controversies in Translating Findings Into Clinical Practice

Translating research findings into clinical practice encounters several challenges and controversies, underscoring the complexity of this process. Despite its importance, numerous barriers impede the incorporation of new evidence into clinical settings, with individual-level issues taking precedence over organisational factors [22]. Individual attitudes, beliefs, knowledge, organisational structures, and processes significantly translate research findings into clinical practice. Barriers to research translation encompass a need for more research education, leading to disinterest, motivational challenges, and difficulties in interpreting and applying research findings in clinical settings [23,24]. These obstacles threaten the seamless integration of research evidence into clinical care. A consistent observation in clinical and health services research is the failure to translate research into practice and policy. This results in suboptimal patient benefits, increased risks of iatrogenic harm, and substantial opportunity costs for healthcare systems [25]. To overcome these barriers, the establishment of collaborations and partnerships between policymakers and health professionals at all levels and stages of the research process is paramount [23]. Effective stakeholder collaboration and cooperation are crucial for enhancing the translation of research findings into clinical practice. Recognising the challenges and facilitators is essential for prioritising key initiatives that facilitate the translation and integration of research evidence into practice globally.

Potential mechanisms of action in sepsis

Regulation of Inflammation

The dysregulated inflammatory response is a hallmark characteristic of sepsis, playing a pivotal role in the condition's pathophysiology and contributing significantly to tissue damage and organ dysfunction. Vitamin D emerges as a potential modulator of this inflammatory cascade, offering a unique avenue for intervention. Central to Vitamin D's role in sepsis is its capacity to finely regulate inflammation. Vitamin D aims to mitigate the exaggerated pro-inflammatory response often observed in sepsis through its influence on various immune cells and cytokine production [26]. Studies indicate that Vitamin D regulates immune cells, suppressing the production of key pro-inflammatory cytokines, including interleukin-6 (IL-6) and tumour necrosis factor-alpha (TNF-α). Simultaneously, Vitamin D promotes the synthesis of anti-inflammatory cytokines, such as interleukin-10 (IL-10). This dual action is crucial for restoring immune homeostasis and fostering a balanced immune environment. By dampening excessive inflammation and promoting an anti-inflammatory milieu, Vitamin D strives to prevent the progression of sepsis to more severe stages [27]. The regulatory effect of Vitamin D on inflammation is particularly significant in the context of sepsis, where an uncontrolled and dysregulated immune response can lead to systemic consequences. By modulating the cytokine profile, Vitamin D may help prevent the cytokine storm associated with severe sepsis, ultimately reducing the risk of organ dysfunction and mortality. This nuanced approach to immune modulation positions Vitamin D as a promising adjunct in managing sepsis, offering potential benefits in mitigating the inflammatory cascade and improving patient outcomes. Further research is warranted to elucidate the optimal strategies for incorporating Vitamin D into sepsis management protocols and to determine its efficacy in diverse patient populations [28].

Enhancement of Antimicrobial Responses

Vitamin D is pivotal in fortifying the innate immune system's antimicrobial responses, offering a compelling mechanism for potential therapeutic intervention, especially in sepsis. One of the fundamental mechanisms through which Vitamin D enhances antimicrobial defences is the induction of antimicrobial peptides, with cathelicidin standing out prominently in this regard. Cathelicidin, induced by Vitamin D, exhibits potent antimicrobial properties and plays a crucial role in the clearance of pathogens [29]. In the complex landscape of sepsis, where the underlying infection often dysregulates the immune response, the capacity to fend off microbial invaders becomes compromised. Vitamin D's enhancement of antimicrobial responses becomes particularly relevant in this scenario. By supporting the innate immune system's ability to eliminate pathogens, Vitamin D may contribute significantly to the resolution of infection and, importantly, aid in preventing sepsis-related complications [3]. The induction of cathelicidin by Vitamin D represents a strategic approach to bolstering the host's defences against microbial threats. In sepsis, where the risk of uncontrolled infection and systemic dissemination is heightened, this augmentation of antimicrobial responses holds promise for influencing the course of the condition. Further exploration of the specific mechanisms underlying Vitamin D's impact on antimicrobial peptides and its potential to improve sepsis outcomes is crucial. Incorporating Vitamin D as a modulator of antimicrobial responses may present an innovative avenue for therapeutic strategies, offering a targeted approach to addressing the infectious component of sepsis and contributing to the overall management of this complex clinical syndrome [30].

Impact on Organ Dysfunction and Mortality

The dysregulated immune response in sepsis not only contributes to systemic inflammation but also plays a pivotal role in the development of organ dysfunction and, ultimately, mortality. Vitamin D's multifaceted mechanisms of action, including its influence on immune cell function and cytokine balance, position it as a potential modulator of organ dysfunction in sepsis. Vitamin D may positively impact the course of organ dysfunction by mitigating excessive inflammation, promoting a balanced immune response, enhancing antimicrobial defences, and improving patient outcomes [14]. Furthermore, emerging evidence suggests a potential link between Vitamin D deficiency and increased mortality in sepsis patients. Addressing Vitamin D status may thus represent a novel avenue for intervention. However, it is crucial to note that the clinical translation of these potential mechanisms into effective therapeutic strategies requires further investigation, considering the complexity of sepsis and the need for comprehensive, patient-tailored approaches [18].

Challenges and considerations

Variability in Vitamin D Status and Its Measurement

One of the primary challenges in exploring the role of Vitamin D in sepsis is the inherent variability in individuals' Vitamin D status. Factors such as geographic location, season, skin pigmentation, and dietary habits contribute to significant variations in Vitamin D levels among diverse populations. Moreover, measuring Vitamin D status poses challenges, with different assays and thresholds for deficiency complicating the interpretation of study findings. Addressing these variations is crucial for accurately assessing the impact of Vitamin D on sepsis outcomes and tailoring interventions based on individual needs [18].

Optimal Dosages and Duration of Supplementation

Determining the optimal dosage and duration of Vitamin D supplementation in sepsis is complex. While studies suggest the potential benefits of Vitamin D supplementation in various diseases, including sepsis, there has yet to be a consensus on the most effective dosages and treatment durations. The lack of standardised guidelines hinders the translation of research findings into clinical practice. Understanding the appropriate balance between achieving therapeutic effects and avoiding potential adverse outcomes is essential for establishing evidence-based recommendations for Vitamin D supplementation in sepsis [31].

Heterogeneity in Patient Populations

Sepsis is a heterogeneous syndrome with diverse underlying infections, patient demographics, and comorbidities. This heterogeneity poses a challenge when studying the effects of Vitamin D, as responses may vary across different patient populations. Factors such as age, comorbidities (e.g., diabetes, cardiovascular disease), and the nature of the underlying infection can influence both Vitamin D metabolism and the immune response. Recognising and accounting for this heterogeneity is essential to draw meaningful conclusions about the efficacy of Vitamin D interventions in sepsis [32].

Potential Adverse Effects and Toxicity

Despite the generally recognised safety of Vitamin D, a thorough consideration of potential adverse effects and toxicity is essential, particularly in the specific context of sepsis. A cautious approach is warranted due to the intricate interplay between Vitamin D and various physiological processes, especially in critically ill patients [33]. High doses of Vitamin D supplementation, although aiming to address deficiencies and modulate immune responses, may carry the risk of inducing hypercalcaemia. Elevated levels of calcium in the blood can have detrimental effects on renal function and cardiovascular health, potentially exacerbating complications in septic patients who may already be experiencing organ dysfunction. Given the delicate balance of electrolytes and organ homeostasis, the potential consequences of hypercalcaemia require careful consideration in the septic population [33]. Understanding the risk-benefit profile of Vitamin D supplementation in septic patients, especially those with pre-existing conditions, is crucial for patient safety. The severity of sepsis and comorbidities may influence the body's response to Vitamin D, necessitating individualised approaches to supplementation. It is imperative to weigh the potential benefits of Vitamin D in modulating the immune response against the risks of adverse effects, particularly in critically ill individuals [33]. Meticulous monitoring of Vitamin D levels, calcium concentrations, and relevant clinical parameters is essential in the septic population to detect and manage potential adverse effects promptly. Individualised dosing strategies, guided by careful assessment of patient status, can help balance optimising Vitamin D levels and minimising the risk of adverse outcomes [33].

Future directions

Areas for Further Research

Continued exploration of Vitamin D's role in sepsis warrants in-depth investigation into specific areas. Research should investigate the underlying molecular mechanisms that Vitamin D influences immune cells, cytokines, and immune responses during sepsis. Understanding the impact of Vitamin D in different stages of sepsis and its interaction with other therapeutic modalities would provide valuable insights. Additionally, investigating Vitamin D's potential as a predictive marker for sepsis outcomes and severity could guide early intervention strategies [21].

Clinical Implications and Potential Interventions

Translating research findings into clinical practice requires a comprehensive understanding of Vitamin D's clinical implications in sepsis. Longitudinal studies assessing Vitamin D status in septic patients and its correlation with clinical outcomes are crucial. Moreover, investigating the potential benefits and risks of Vitamin D supplementation in diverse patient populations, including those with varying degrees of Vitamin D deficiency, is essential for informing evidence-based clinical interventions [19].

Development of Targeted Therapies Based on Vitamin D Modulation

The identification of specific pathways influenced by Vitamin D opens avenues for the development of targeted therapies. Investigating the feasibility and efficacy of tailored Vitamin D interventions, such as dose optimisation and administration routes, is critical. Moreover, developing novel therapeutic agents that mimic or enhance the immunomodulatory effects of Vitamin D could pave the way for more precise and effective treatments for sepsis. Collaborative efforts between researchers, clinicians, and pharmaceutical entities are essential for advancing these potential targeted therapies from bench to bedside [33].

## Conclusions

In conclusion, this comprehensive review has illuminated the multifaceted role of Vitamin D in modulating the immune response in sepsis. The key findings underscore Vitamin D's ability to regulate immune cell function, modulate cytokine production, and enhance antimicrobial responses. However, challenges such as the variability in Vitamin D status, uncertainties regarding optimal dosages and patient heterogeneity, and potential adverse effects necessitate a cautious interpretation of these promising results. Looking ahead, the implications for future research and clinical practice are substantial. Standardising measurement protocols, elucidating optimal supplementation strategies, and integrating Vitamin D assessments into routine care could pave the way for personalised interventions in septic patients. While Vitamin D's potential in sepsis is evident, ongoing research collaborations and evidence-based guidelines are essential to unlock its full therapeutic promise and translate findings into improved clinical outcomes.
